# Substance Use Disorders and Psychiatric Comorbidity

**DOI:** 10.1192/j.eurpsy.2026.10810

**Published:** 2026-07-31

**Authors:** K. Taleb, F. Laajili, H. Ballouk, Z. Bencharfa, F. El Omari

**Affiliations:** 1 Psychiatry; 2 Arrazi Hospital, Salé, Morocco

## Abstract

**Introduction:**

Substance use disorders (SUDs) are often associated with other mental disorders—psychotic, mood, or anxiety—complicating treatment and worsening clinical and social outcomes.

**Objectives:**

To describe the main psychiatric comorbidities among patients with SUDs and examine their relationship with substance type.

**Methods:**

A retrospective descriptive study based on clinical records of patients consulting for SUDs. Collected data included sociodemographic profile, substances used, psychiatric diagnoses, and treatments.

**Results:**

**Results (N = 102):**

- Gender: predominantly male (~90%).
- Age: mostly 20–40 years.
- Main substances: tobacco (85%), cannabis (70%), alcohol (56%).
- Comorbid disorders: psychotic (45%), mood (30%), anxiety (15%), personality (10%).
- Polysubstance use: >50% of patients.
- Treatments: antipsychotics (79%), antidepressants (55%), anxiolytics (38%).

**Conclusions:**

SUDs are commonly associated with severe psychiatric comorbidities. Systematic screening and a dual-diagnosis approach are key to enhancing prognosis and patient reintegration.

**Disclosure of Interest:**

None Declared

